# Supplementary material for: Integrative review in PhD admissions: A case study of efficiently minimizing bias while maximizing the student narrative
Source: PLoS One. 2025 Jun 16;20(6):e0323426. doi: 10.1371/journal.pone.0323426 (PMC12169560; doi:10.1371/journal.pone.0323426)
Supplement: Attachment — Submitted filename: Response to Reviewers_Rev2.docx [file pone.0323426.s003.docx]

**The authors thank the Reviewers again for their continued comments that have greatly benefitted this manuscript. The suggestions have been integrated into this second revision. If relevant, line numbers reflecting edits specific to each reviewer comment have been included.**

Reviewer #1:

The authors have significantly improved the paper, and I commend them for their efforts to address all of the previous comments. I only have two comments that I think are truly vital to be addressed before the paper is ready for publication.

1. The authors analyzed the odds of a student reaching a particular phase in the admissions process given certain characteristics (e.g. URM status), which makes the data much easier to interpret than in the previous version of the paper. However, the discussion of these results is very brief. The paper (and its readers!) would benefit substantially from a subsection in the Discussion talking about the implications of these results and spelling out the conclusions for the readers.

**Thank you for this critique. The results sections describing the data (Lines 222-228 and 230-239) and the discussion (Lines 263-282) have been edited to expand our interpretation of these results and the implications have been further detailed later in the discussion (Lines 297-313).**

2. I still find myself somewhat unsure what the reader is meant to learn from this data so that they can improve their university's admissions processes. However, I have a much better guess from this version of the paper due to the authors' significant improvements. Is the take-home message that the de-identified PS evaluation produces the same results as an algorithmic admissions procedure, even though it takes significantly less time to perform? If that is the case, the authors should state that explicitly and present their case study as evidence for using de-identified personal statements to decrease the amount of time required for the first round of application reviews. Otherwise, I am not sure what conclusions I am meant to draw as a reader.

**The authors appreciate the perspective shared by the reviewer. To more directly state the purpose of this process and its key takeaways, we have added a section to the discussion (Lines 307-325) as well as edited the conclusion to provide a more cohesive and consistent message (Lines 373-378). The authors hope that these (and other changes made in this revision) have helped to hone in on the central point that this type of process promotes the prioritization of the student’s own narrative while minimizing potential bias.**

There is one additional thing I would like to see addressed that is less crucial but would help support the overall conclusions of the paper, assuming that I am now interpreting the paper correctly. What was the variance on the 7 application reviewers' scores of the de-identified PSs? I am concerned that most graduate programs would not be able to support having 7 reviewers for each de-identified PS. Is it the case that 7 reviewers were actually needed to come to a consensus, or could a consensus reasonably have been reached with (say) 3 reviewers? Knowing the answer to this question could help the authors discuss how widely applicable their case study may be.

**The authors appreciate the opportunity to address this point to clarify the conclusions of the paper, Variance cannot be determined as rubrics were used to gather qualitative feedback – this has been clarified in the methods section (Lines 158-178). The question of how many reviewers are needed to reach consensus is a good one, our process includes multiple program stakeholders to participate in the admissions committee. Although we cannot recommend a specific number, we have included more specific details about the application review process, including the makeup of the 7 reviewers for clarity (Lines 163-164). In the limitations, we have expanded on the issue of resource intensity/variance to suggest approaches that may be more widely applicable (Lines 357-362).**

Reviewer #2:

I am grateful to the reviewers for a thorough revision of the manuscript, taking on board the range of points raised and critiques of the limitations of evidence that can be drawn from the case study that this effectively presents. I have a few remaining points that I think would be helpful to address in order to maximise the value of this paper to readers, as well as a few minor points that I spotted while reading.

The short title of the paper and its full title are quite strikingly different. Both have their merits so I think it’s worth reflecting on what contribution the paper is making that you want to foreground in the title. For me, showcasing an example of an integrative application review process is definitely part of it. I would suggest something like “Integrative application review in graduate school PhD admissions: a case study of efficiently minimizing bias while maximizing the student narrative” as the full title, with a short title that drops the bits after the colon, but that’s just one fairly quick go at that.

**Thank you for this insightful comment. The short and full titles have been edited accordingly. (Lines 1-3 and Headers)**

Drawing somewhat on an embedded point of that last one, I think you could be more explicit that this is, effectively, a case study. Case studies have strengths and limitations and thinking of the study in this way will help people interpret the results appropriately, noting that this will imply attention to context in seeking lessons for other contexts.

**Per the suggestion to more appropriately label this work a case study, references to clarify the study type have been made throughout (Lines 1, 25, 150, 253, 337, 339, 374-375). A discussion of the limitations of this work being a case study has been added to the limitations (Lines 336-342)**

Relatedly, the discussion section could helpfully do more to foreground the implications of the paper’s finding for others running higher education admissions exercises (especially graduate biomedical programmes, although perhaps not exclusively). In service of drawing out such implications, you could link your findings to other areas of the literature on higher education admissions that have sought to understand how aspects of the admissions process affect potential biases. (Full disclosure: I wrote the paper I’m about to mention and you should not feel under any obligation to cite it but, perhaps inevitably, connections to one’s own work will come to mind!) Anders (2014) found that introducing an aptitude test in the admissions process for a highly competitive undergraduate course reduced the number of applicants who needed to be interviewed without affecting gender or socioeconomic representation in those selected, which mirrors your own finding of being able to streamline admissions using your proposed framework without negatively affecting diversity. There are other papers that have relevant findings against which yours could be compared, likely with closer context.

**The authors appreciate the suggestion. A paragraph discussing implications for similarly minded program administrations has been added to the discussion and includes references to similar efforts/results (Lines 307-313).**

The paper’s conclusions has become a little disjoint from the rest, where the focus has become more on the overall nature of the ‘integrative’ application review process, while this just mentions blinded review in isolation. I think having embedded it within a wider framework has been helpful in explaining what you have done and its potential lessons, so would encourage you to ensure the conclusion section aligns with this (potentially also checking for similar issues elsewhere).

**Thank you for this comment. The authors have edited the manuscript throughout to sharpen the focus to ‘integrative review using blinding’ rather than interchanging the terms blinded review and integrative review. As previously, the conclusion has been revised to provide better overall clarity and cohesion (Lines 373-378).**

Minor point: Claims in the introduction/review sections are mostly well-evidenced but the sentence “We agree with prior cautions that this style is most prone to biases that may limit desired demographic changes and equitable access for all applicants.” on lines 117-119 would benefit from a citation to help people trace evidence of the type discussed.

**Thank you for this catch. A citation to support this evidence has been added (Line 120).**
